# Dysbiosis of Gut Microbiota in Microscopic Colitis: Diagnostic and Therapeutic Implications

**DOI:** 10.3390/diagnostics15141733

**Published:** 2025-07-08

**Authors:** Sanja Dragasevic, Andreja Nikolic, Sanja Zgradic, Milica Stojkovic Lalosevic, Stefan Stojkovic, Vera Matovic Zaric, Snezana Lukic, Tijana Glisic, Stefan Kmezic, Dusan Saponjski, Dragan Popovic

**Affiliations:** 1 Clinic for Gastroenterology and Hepatology, University Clinical Center of Serbia, Koste Todorovica 2, 11000 Belgrade, Serbia; andrejanikolich@gmail.com (A.N.);; 2 Faculty of Medicine, University of Belgrade, Dr Subotica 8, 11000 Belgrade, Serbia; 3 Clinic for Digestive Surgery, University Clinical Center of Serbia, Koste Todorovica 6, 11000 Belgrade, Serbia; 4 Center for Radiology Imaging-Magnetic Resonance, University Clinical Center of Serbia, 11000 Belgrade, Serbia

**Keywords:** microscopic colitis, gut microbiota, dysbiosis, metagenomics, inflammatory bowel disease, microbial diversity, *Akkermansia muciniphila*, *Veillonella dispar*

## Abstract

Microscopic colitis (MC) is an idiopathic inflammatory bowel disease characterized by watery, non-bloody diarrhea and histopathological changes but normal endoscopic findings. Increasing evidence now suggests that alterations in the gut microbiota contribute to the pathogenesis of MC. In this narrative review, we summarize evidence from nine case-control studies examining microbial composition using sequencing technology. The research presented here illustrates reduced alpha diversity, high dysbiosis, and pro-inflammatory oral-associated taxa enrichment, such as *Veillonella dispar*, and loss of protective microbes such as *Akkermansia muciniphila* and *Bacteroides stercoris*. These microbial changes have the potential to be non-invasive diagnostic biomarkers that can differentiate MC from other etiologies. In addition, the characterization of gut microbiota in MC can guide personalized therapeutic strategies, such as directed probiotic therapy or fecal microbiota transplantation, to help restore microbial balance. These microbial patterns can be applied to guide the creation of diagnostic biomarkers and personalized therapy. Despite differences in sample types and sequencing methods, general microbial trends highlight the need for further longitudinal and standardized investigations.

## 1. Introduction

Microscopic colitis (MC) is an inflammatory bowel disease that is characterized by chronic diarrhea without blood, endoscopically normal mucosa of the colon, and pathognomonic histopathology. Two main subtypes of MC are currently recognized: collagenous colitis (CC), identified by subepithelial collagen bands exceeding 10 μm in thickness, and lymphocytic colitis (LC), which is characterized by evidence of more than 20 intraepithelial lymphocytes/100 epithelial cells in colon biopsies [1,2].

Recent epidemiological data indicate an increased incidence and prevalence of MC on a worldwide level, indicating an increased need for the elucidation of underlying pathophysiological mechanisms [3]. Despite improved clinical diagnosis over the years, certain etiologic causes and detailed pathogenic mechanisms of MC remain to be elucidated fully, particularly the potential role of the gut microbiome. Because fecal diversion procedures have a strong influence on MC disease activity—suggesting a critical role of gut microbiota—the identification of specific microbial alterations associated with MC pathogenesis can offer new diagnostic biomarkers and targeted therapeutic strategies [4,5,6].

Previous studies have explored gut microbiota disturbances in MC and reported numerous microbial alterations, including decreased microbial diversity, increased microbial dysbiosis, and varied abundance of some bacterial taxa such as *Akkermansia muciniphila*, *Veillonella dispar*, and Ruminococcaceae and Coriobacteriaceae family members [5,6,7,8,9,10,11]. These studies are often undermined by methodological heterogeneity, small numbers of samples, and conflicting results, which disallow the drawing of firm conclusions on microbiota composition in MC [7].

In light of existing knowledge gaps and methodological restrictions in the field, our primary objective with this review was to critically assess all available high-quality case-control studies that investigated changes in gut microbiota in microscopic colitis. Importantly, this update integrates recent far-reaching evidence, namely the large and rigorously designed study of Chen et al. (2024) [6], thereby increasing the scope and relevance of our overview. We aimed to briefly overview microbial patterns robustly associated with MC, evaluate their potential diagnostic and therapeutic implications, and outline important avenues for further investigation. This article represents a narrative review, integrating available high-quality case-control studies on gut microbiota in microscopic colitis without adhering to formal systematic review protocols.

Studies on microscopic colitis have used biopsies or fecal samples for obtaining microbial assessment [7]. The microbiome was analyzed using sequencing technology in MC. The majority of research studies used sequencing methods focused on 16S ribosomal RNA (rRNA) or 16S ribosomal DNA (rDNA) [7]. Nevertheless, some investigations applied metagenomic sequencing. Microbial culture is a less commonly used culturing technique that can identify specific bacterial species [7].

The alpha diversity, commonly measured by the Shannon index, was significantly lower in MC compared to healthy controls [7,8]. However, some studies showed no variations between groups [9]. No differences were identified for the beta diversity [7]. Higher values of microbial dysbiosis index were detected in active MC compared to the remission phase of MC [10]. In addition, no differences were registered between CC and LC regarding alpha diversity, beta diversity, and microbial dysbiosis index [11]. Furthermore, there were no differences in the alpha diversity between MC and controls with diarrhea (DC) (Crohn’s disease, ulcerative colitis, functional DC, and bile acid DC) [11].

Batista L. et al. conducted a study demonstrating that microbial diversity is associated with stool form [11]. This study highlighted the effect of colonic lavage after a diagnostic colonoscopy on microscopic colitis. Furthermore, several studies on the microbiome in MC have been conducted after endoscopic procedures. Colonic preparations based on polyethylene glycol eliminate mucus and endoluminal bacteria, resulting in microbiota imbalance. Introducing oxygen into the colonic lumen during preparation can adversely impact anaerobic bacterial populations. Reduction of available nutrients, including fiber and other fermentable carbohydrates, along with accelerated intestinal transit, can contribute to disturbed microbiota homeostasis [12].

According to published data, various microbes have been identified as significant contributors to the pathogenesis of collagenous colitis, including *Clostridioides difficile*, noroviruses, Escherichia species, *Campylobacter concisus*, and *Campylobacter jejuni* [12,13]. This can be explained by a decrease in protective bacteria, such as *Lactobacillus* spp., *Bifidobacterium* spp., and *Akkermansia muciniphila*, as well as by microbes producing pro-inflammatory cytokines [1,14]. Fasullo M.J. et al. reported cases of MC occurrence after fecal microbiota transplantation (FMT) [15]. This research proposes that inflammation-inducing lymphocytes migrate into the gut after FMT into a host with impaired immune function. Furthermore, an abrupt increase in bacterial diversity can elevate bacterial metabolite levels, potentially contributing to the development of microscopic colitis [15].

## 2. Literature Search and Selection Strategy

### 2.1. Selected Case-Control Studies

As part of this narrative review, we performed a non-systematic search of PubMed up to April 2025 using the keywords: ‘microscopic colitis’, ‘gut microbiota’, ‘dysbiosis’, ‘metagenomics’, and ‘microbial diversity’. We searched PubMed for relevant papers published up to April 2025 with the search terms ‘microscopic colitis’, ‘gut microbiota’, ‘dysbiosis’, ‘metagenomics’, and ‘microbial diversity’. In this review, we preferred case-control studies that compared the gut microbiota composition in adult microscopic colitis patients with controls of either healthy subjects or patients with diarrhea and employed sequencing-based methods (16S rRNA or whole-genome shotgun metagenomics).

For inclusion, studies needed to be original articles in English between 2015 and 2025 published in peer-reviewed journals and contain sufficient methodological detail to allow microbiota analysis. Being able to report a Newcastle-Ottawa Scale score ≥ 6 [16] was taken as a sign of greater methodological quality but not an exclusion criterion per se, rather a precaution to help our consideration of study quality in discussion. Reviews, editorials, letters, case reports, and studies without clear microbiome data were not considered.

No studies meeting these inclusion criteria were excluded on any unstated grounds. The process was selective and narrative in nature, attempting to present an integrated but focused overview of available evidence.

Ultimately, nine case-control studies were included and presented in Table 1.

Although the total number of studies included in our narrative review seems small, it is important to note that these nine studies represent all currently published literature identified on PubMed on microbiome composition in microscopic colitis fulfilling the strict quality criteria. The extension of the analysis for the incorporation of lower methodological quality studies would necessarily compromise the quality and soundness of conclusions drawn. In addition, the inclusion of the just-completed extensive survey of Chen et al. [6] enriches the present review significantly by offering valuable new information apart from reaffirmation of previously found microbial trends. This manuscript was prepared using Microsoft Word for Windows, Microsoft 365 (Microsoft Corp., Redmond, WA, USA).

### 2.2. Overview of Study Design and Participant Population

Our paper comprised nine case-control studies that reported data on 314 MC patients. Specifically, Chen et al. (2024) reported a large cohort of 131 active MC patients with two distinct control groups: one comprising patients with chronic diarrhea (*n* = 159) and the other comprising age- and sex-matched controls with no diarrhea [6]. The other studies in our review reported data on 183 MC patients with healthy controls (*n* = 121) as the most frequent reference group. Other control groups consisted of patients with bile acid diarrhea (*n* = 16), functional diarrhea (*n* = 31), Crohn’s disease (*n* = 53), and ulcerative colitis (*n* = 70) [5,8,9,10,11,18,19,20]. All the studies targeted the adult population.

Four studies examined MC subtypes, while the Carstens A. et al. study consisted exclusively of CC patients [17]. Autoimmune disorder and previous budesonide treatment in MC patients were examined by Carstens A. et al. [17], while the Krogsgaard et al. study involved MC patients who were treated with budesonide after their initial microbiome assessment [18].

Previous and current smoking was identified as a key predictor of microbiome composition in four of nine highlighted case-control studies [9,10,19,20]. Four studies also reported on BMI status and revealed a broad weight profile in MC patients [6,10,11,18]. Most studies included in the present review imposed a 3-month or 1-month washout period for antibiotics and probiotics before inclusion.

Sampling for microbial analysis was performed on feces and tissue biopsy samples. Whereas Fischer et al., Morgan et al., and Chen et al. employed whole-genome shotgun metagenomic sequencing [6,10,19], the rest utilized 16S rRNA or 16S rDNA amplification procedures for microbial community characterization. The majority of the included studies using 16S rRNA sequencing targeted the V3–V4 or V4 hypervariable regions. Bi Bioinformatic analysis predominantly involved standard pipelines such as QIIME (Quantitative Insights Into Microbial Ecology), mothur (bioinformatics pipeline), or DADA2 (bioinformatics pipeline) for taxonomic classification [5,8,9,11,18,19].

## 3. Microbial Signatures in Microscopic Colitis

Chen A.S. et al. detected a significant shift in the microbial and metabolomic composition of the stool in MC [6]. The study indicated that the gut microbiome in MC patients is characterized by lower alpha diversity, enriched pro-inflammatory oral-typical species (*Veillonella dispar* and *Haemophilus parainfluenzae*), and depleted anti-inflammatory beneficial microbes (*Blautia glucerasea* and *Bacteroides stercoris*) [6]. Furthermore, the gut metabolome in MC patients expressed a significant enrichment of proinflammatory metabolites (lactosylceramides, ceramides, lysophospholipids, and lysoplasmalogens) [6]. Multi-omics investigations highlight robust and consistent relations between microbes, metabolic pathways, and metabolomic profiles [6].

Studies on alpha and beta diversity in MC patients have yielded mixed results. While some investigations found no differences between MC patients and healthy controls, others reported a significantly lower alpha diversity in MC patients than in healthy individuals. Consistent with these findings, Chen et al. reported that active MC was characterized by reduced alpha diversity compared to both controls and MC in remission [6]. Notably, Chen et al. observed an increase in alpha diversity following budesonide treatment, suggesting a possible partial restoration of the microbiota. However, this finding remains strictly correlational rather than causal, considering potential confounding influences such as concurrent dietary modifications, improved clinical status, medication adherence, and reduced inflammatory activity. Additionally, they observed distinct clustering between active MC and control groups, indicating shifts in community structure associated with disease activity [6].

Deep taxonomic analysis of MC reported similar trends in studies, i.e., the increased relative oral-typical, pro-inflammatory taxon abundance, most prominently of the genus Veillonella including *V. dispar* and *V. parainfluenzae* [6,8,10,20]. In addition, a reduction of anti-inflammatory bacteria such as *Blautia glucerasea* and *Bacteroides stercoris*, and probiotic microorganisms such as *Akkermansia* (phylum Verrucomicrobia) has been previously described in MC by numerous studies [6,7]. Millien V. et al. and Hertz S. et al. demonstrated a reduced family Coriobacteriaceae in the biopsy and fecal samples [8,9]. Both the Ruminococcaceae (Ruminococcaceae unclassified, *Ruminococcus* 1 and 2 genus, NK4A214, UCG-002, UCG-005, and UCG-010 groups) and Coriobacteriaceae (Coriobacteriaceae unclassified, *Collinsella* genus) families were found to be reduced in MC patient fecal samples [7]. Conversely, the Ruminococcaceae family was found to rise from baseline after treatment with budesonide, according to a study by Krogsgaard et al. [18].

Overall, the common patterns, including the enrichment of pro-inflammatory taxa and depletion of beneficial microbes in MC were largely concordant across the studies, with the recent findings by Chen et al. reinforcing these observations [6]. These results underscore the complex alterations in microbial composition associated with MC and support the potential for microbiome-based biomarkers in their diagnosis and monitoring. The variations in microbial diversity and significant taxonomic differences among MC, healthy controls, and diarrhea controls are summarized in Table 2.

**Figure 1:**
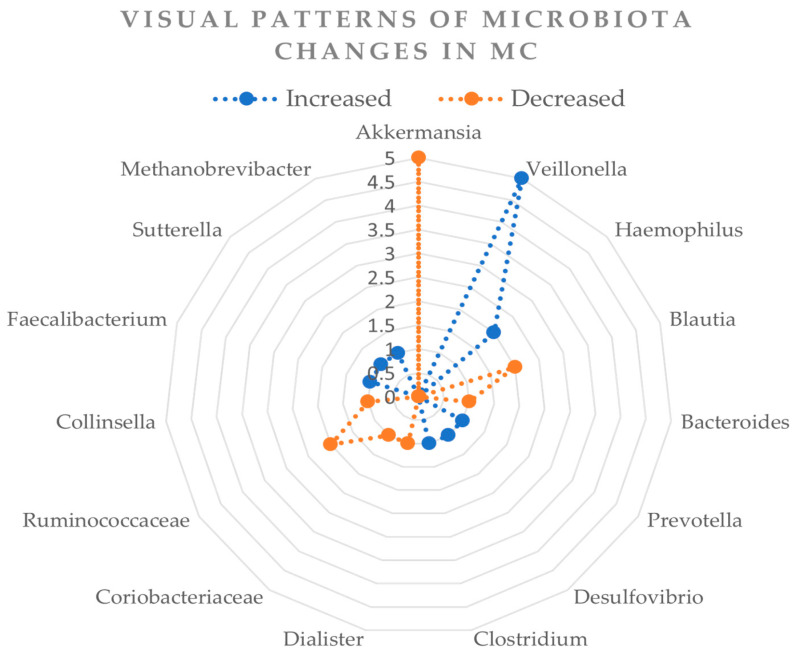
Visual summary of consistently altered bacterial taxa in microscopic colitis. The numbers represent how many studies reported a significant increase or decrease in the respective bacterial taxa among patients with microscopic colitis compared to healthy controls.

Additionally, we created an additional visual chart summarizing the changes in bacterial taxa across all included studies. This clear visual representation highlights patterns of consistently increased or decreased bacteria in microscopic colitis (Table 3). The numbers presented in the chart represent the number of studies in which each bacterial taxon was found to be significantly increased (“Increased”) or significantly decreased (“Decreased”) in microscopic colitis patients compared to healthy controls (Figure 1). No significant difference in abundance across most studies was observed for certain taxa, including *Methanobrevibacter*, *Sutterella*, *Faecalibacterium*, *Collinsella*, *Dialister*, *Clostridium*, *Desulfovibrio*, *Prevotella*, and *Bacteroides* [8,9,11,17,18]. Their role remains unclear or less consistent in MC pathogenesis.

## 4. Discussion

### 4.1. Summary of Main Findings

Our narrative review revealed consistent microbial alterations in MC, such as reduced alpha diversity, increased microbial dysbiosis, loss of protective anti-inflammatory bacteria (e.g., *Akkermansia muciniphila*, *Blautia glucerasea*, and some Ruminococcaceae species), and enrichment with pro-inflammatory, oral-associated taxa *Veillonella dispar* and *Veillonella parainfluenzae* [5,6,8,10,11]. The microbial alterations are most likely central to MC pathogenesis by compromising gut barrier integrity and promoting intestinal inflammation [1,7].

### 4.2. Comparison with Previous Research

The microbial patterns found in our review concur with the recently published large-scale study of Chen et al. (2024) [6], and with earlier observations by Sun et al. (2022) [5], Hertz et al. (2021) [8], Batista et al. (2022) [11], and Morgan et al. (2020) [10]. Chen et al. also significantly highlighted reduced alpha diversity and increased dysbiosis indexes during active MC compared to remission, in concordance with our findings [6]. Variations between other studies—e.g., in beta diversity and taxonomic differences—may be attributed to methodological variations including the utilization of different sequencing approaches (16S rRNA vs. whole-genome shotgun metagenomics) and the character of biological samples used (fecal vs. biopsy samples) [5,6,7].

### 4.3. Methodological Considerations and Limitations

Although this review did not follow a systematic protocol, we carefully selected and synthesized relevant studies of sufficient methodological quality to draw meaningful insights. The methodological diversity of the reviewed studies is a significant disadvantage. More particularly, sample type (feces or biopsy) has a tremendous impact on microbial profiling because the mucosal microbiota is better reflected by biopsies [7]. Furthermore, while 16S rRNA gene sequencing is prevalent, whole-genome shotgun metagenomics has higher resolution and may account for part of the dissimilarities highlighted by studies [6,10]. In particular, biopsy tissue directly acquires mucosal microbiome states, tends to more accurately represent inflammatory changes than fecal samples, which are primarily indicative of luminal bacteria and may be influenced by transit time, diet, and specimen handling. Likewise, whole-genome shotgun sequencing, by having greater sequencing depth and taxonomic resolution, might capture subtle microbial differences beyond the reach of traditional 16S rRNA gene sequencing and, therefore, significantly contributes to observed inter-study variability. Our review only included high-quality studies (Newcastle-Ottawa Scale ≥ 6), thus limiting the overall number of included studies. Although this restriction encourages methodological rigor, it also underscores the need to perform additional well-designed studies to increase generalizability.

While we acknowledge that smoking, BMI, and medication use are key potential confounders influencing microbiome composition, systematic control for these factors varied significantly across included studies. This variability presents a notable limitation. Future studies should standardize approaches for controlling these confounders—ideally through matched study designs or rigorous multivariate statistical adjustment—to better isolate the effects of microscopic colitis on gut microbiota.

This variability presents a notable limitation. Future studies should standardize approaches for controlling these confounders—ideally through matched study designs or rigorous multivariate statistical adjustment—to better isolate the effects of microscopic colitis on gut microbiota. A summary of the main limitations of the currently available studies is presented in Table 3.

### 4.4. Diagnostic and Therapeutic Implications

Our findings have significant clinical implications and suggest the possibility that gut microbiota can be a promising diagnostic and therapeutic target in MC. Therapeutically, the reconstitution of microbiome diversity—potentially with the administration of targeted probiotics, diet, or fecal microbiota transplantation (FMT)—is a new potential answer, but caution is indicated by potential adverse immune responses reported in some FMT treatments [15]. Probiotic supplementation represents a potential therapeutic approach in microscopic colitis to rebalance the microbes, enhance the gut barrier function, and inhibit inflammation. Probiotic efficacy data, however, in the particular situation of MC are limited [15]. Concurrently, fecal microbiota transplantation (FMT) has shown therapeutic promise through the rapid reconstitution of microbial diversity but with serious limitations like the risk of adverse immune reactions and unpredictable long-term outcomes in MC patients [15].

Future research would best be conducted using longitudinal multi-omic study designs with standardized methods to rigorously establish the causative role of changes in the microbiota for MC. Exploring microbial metabolomic profiles, host–microbe interactions, and tailored microbiome-based interventions could significantly improve disease control [6,7,15].

Recent research has found reproducible alterations in MC patient gut microbiota, including the depletion of *Akkermansia muciniphila* and *Veillonella dispar* enrichment, that are promising non-invasive biomarkers for MC diagnosis. Microbiome-derived signatures can help distinguish MC from other etiologies of chronic diarrhea, including irritable bowel syndrome or inflammatory bowel disease.

Furthermore, the determination of particular microbial alterations can aid in the identification of subgroups of patients qualifying for microbiome-directed treatment in a personalized fashion, i.e., as particular probiotic preparations or fecal microbiota transplantation. Treatment personalization based on microbial composition is a new paradigm with the potential to increase treatment response rates and limit exposure to ineffective treatment.

To bring these findings to the clinic, multicenter studies and harmonization of sample collection, sequencing, and data analysis protocols will be required. Community sharing of raw sequencing data may facilitate meta-analyses with harmonized analytical pipelines, boosting the reliability of microbiome-based diagnostics.

## 5. Conclusions

Based on this narrative review, gut microbiota dysbiosis plays a central part in MC pathology. Prior reviewed studies demonstrate that MC is marked by profound changes in microbial diversity and composition. Specifically, several studies have shown reduced alpha diversity as well as a rise in the dysbiosis index in active MC, with loss of beneficial taxa as well as enrichment of oral-associated, inflammatory bacteria. These microbiota alterations can compromise the gut barrier as well as trigger inflammatory responses, ultimately bringing on MC’s clinical manifestations.

Differences in methods, sample type (fecal versus biopsy samples), sequencing platform (16S rRNA gene sequencing vs. whole-genome shotgun metagenomics), as well as clinical factors including BMI, diet, and medication intake, are likely to be responsible for some of the discrepancies between studies. Yet, globally, the dysbiosis pattern is similar, indicating that specific manipulation of the gut microbiota can provide new diagnostic and therapeutic strategies. Longitudinal, multi-omic studies employing standardized protocols are required in the future to better highlight the relationship between the gut microbiome and host immune system in MC. Such a strategy holds the potential to guide the creation of personalized microbiome-targeted interventions that aim to optimize disease control.

In summary, while there is compelling evidence for a strong concomitance between dysbiosis of the gut and MC, its use would require additional development and verification. A coordinated, large-scale effort at data harmonization and sharing would be required to utilize microbiota signatures as robust, non-invasive diagnostic and monitoring markers for microscopic colitis.

## Figures and Tables

**Table 1:** Summary of selected case-control studies on microbiome in MC.

Study	Year	Country	Study Population	Control Population (*n*)
Sun S. et al. [5]	2022	USA	53	Unspecified diarrhea (*n* = 153)
Chen et al. [6]	2024	USA	131	Chronic diarrhea (*n* = 159) Controls without diarrhea (*n* = 393)
Hertz S. et al. [8]	2021	Denmark	15	Healthy (*n* = 21) Crohn’s disease (*n* = 21) Ulcerative colitis (*n* = 38)
Millien V. et al. [9]	2018	USA	20	Healthy (*n* = 20)
Morgan D.M. et al. [10]	2020	Sweden	20	Healthy (*n* = 20) Functional diarrhea (*n* = 20)
Batista L. et al. [11]	2022	Spain	16	Healthy (*n* = 14); Bile acid diarrhea (*n* = 16) Functional diarrhea (*n* = 11)
Carstens A. et al. [17]	2019	Sweden	29	Healthy (*n* = 29) Crohn’s disease (*n* = 32) Ulcerative colitis (*n* = 32)
Krogsgaard L.R. et al. [18]	2019	Denmark	20	Healthy (*n* = 10)
Fischer H. et al. [19]	2015	Sweden	10	Healthy (*n* = 7)

Note: This table summarizes data from selected literature sources. It is not part of a systematic review or meta-analysis.

**Table 2:** Overview of microbial diversity and bacterial composition in MC patients across selected studies.

Study	Microbial Diversity in MC Compared to HC	Balance of Gut Bacteria in MC Compared to HC	Bacteria Increased (↑) in MC	Bacteria Decreased (↓) in MC
Sun S. et al. [5]	Reduced diversity	More imbalanced	*Veillonella species*	*Akkermansia muciniphila*
Chen et al. [6]	Reduced diversity	More imbalanced	*Veillonella dispar*, *Haemophilus parainfluenzae*	*Akkermansia muciniphila*, *Blautia glucerasea*, *Bacteroides stercoris*
Hertz S. et al. [8]	Reduced diversity	More imbalanced	*Prevotella copri*, *Veillonella species*	*Blautia*, *Dialister*
Millien V. et al. [9]	No significant difference	More imbalanced	*Desulfovibrio species*	*Coriobacteriaceae family*
Morgan D.M. et al. [10]	Reduced diversity	More imbalanced	*Haemophilus parainfluenzae*, *Veillonella parvula*	*Akkermansia muciniphila*
Batista L. et al. [11]	Reduced diversity	More imbalanced	*Clostridium perfringens*, *Veillonella parvula*	*Ruminococcaceae family*
Carstens A. et al. [17]	No significant difference	No significant difference	*Methanobrevibacter species*	*Collinsella*, *Ruminococcaceae*
Krogsgaard L.R. et al. [18]	No significant difference	No significant difference	*Faecalibacterium*, *Sutterella*	*Akkermansia muciniphila (at baseline)*
Fischer H. et al. [19]	No significant difference	No significant difference	*—*	*Akkermansia muciniphila*

Abbreviations: MC—microscopic colitis; HC—healthy controls; ↑—increased abundance; ↓—decreased abundance; ——no significant bacterial taxa changes observed. All directional changes (increased or decreased bacterial taxa and diversity metrics) represent statistically significant differences (*p* < 0.05) as reported in the original included studies. Note: Information presented is based on qualitative synthesis of findings reported in individual studies.

**Table 3:** Main Methodological Limitations of Current Studies on the Gut Microbiota in Microscopic Colitis.

Limitation	Explanation
Methodological diversity	Different sequencing methods (16S rRNA vs. metagenomics), variable pipelines and thresholds
Small sample sizes	Limited number of MC cases in individual studies
Heterogeneity of populations	Studies include diverse geographic regions, age groups, genders
Lack of unified analytical pipeline	No standardized bioinformatics or taxonomic classification pipeline
Differences in sample types	Some studies use fecal samples, others use biopsies
Limited data sharing	Raw sequencing data often unavailable for meta-analysis
Confounding factors not uniformly controlled	Variations in control for smoking, BMI, medications, and diet

Abbreviations: MC—microscopic colitis; BMI—body mass index; 16S rRNA—16S ribosomal RNA. Note: This visual summary reflects the frequency of specific findings across the included literature and does not represent a statistical meta-analysis.

## Data Availability

No additional data were created during the analysis.
